# A Case of Cerebral Amyloid Angiopathy-Related Inflammation With the Rare Apolipoprotein ε2/ε2 Genotype

**DOI:** 10.3389/fneur.2019.00547

**Published:** 2019-05-24

**Authors:** Yin-Yan Xu, Shuai Chen, Jian-Hua Zhao, Xi-Ling Chen, Jie-Wen Zhang

**Affiliations:** ^1^Department of Neurology, The Second Affiliated Hospital of Zhengzhou University, Zhengzhou, China; ^2^Department of Neurology, Zhengzhou University People's Hospital, Henan Provincial People's Hospital, Zhengzhou, China

**Keywords:** cerebral amyloid angiopathy, inflammation, apolipoprotein gene, homozygote, neuroimaging

## Abstract

Cerebral amyloid angiopathy (CAA)-related inflammation (CAA-RI) is a rare CAA variant characterized by acute or subacute encephalopathy, headache, epilepsy, or focal neurological deficits. Radiologically, CAA-RI presents with widespread white matter lesions on brain magnetic resonance imaging (MRI) in addition to the hemorrhagic imaging features of CAA. Previous studies have found that the apolipoprotein E (*ApoE*) ε4 allele and ε4/ε4 genotype were over-represented in CAA-RI. The role of the *ApoE* ε2 allele in CAA-RI, however, is largely unknown, partly due to the rarity of the ε2/ε2 genotype in the general population. The authors report the first case of CAA-RI with the rare *ApoE* ε2/ε2 genotype. The patient presented with mild clinical symptoms but striking neuroimaging abnormalities. The response to small-dose glucocorticoids was satisfactory. Because *ApoE* ε2 promotes amyloid β accumulation and fibrinoid necrosis in the cerebral vasculature, the ε2/ε2 genotype, similar to ε4/ε4, may also be a precipitating factor for CAA-RI. To clarify the role of *ApoE* ε2 in CAA-RI, studies with large sample sizes investigating whether ε2 is more common in patients with CAA-RI than in those with CAA only are warranted.

## Background

It was not a novel pathological finding that inflammation exists within or surrounding amyloid vessels in cerebral amyloid angiopathy (CAA) (1). Over the past decade, these pathological inflammatory features have corresponded to a special clinical and radiological entity, which is referred as CAA-related inflammation (CAA-RI). Because of its good response to immunosuppressive therapy, CAA-RI has attracted increasing attention. Common manifestations of CAA-RI include acute or subacute onset encephalopathy, headache, epilepsy, and/or focal neurological deficits. Although CAA-RI often presents with severe neurological disturbance(s), other atypical forms of CAA-RI have also been reported including radiologically isolated CAA-RI, minimally symptomatic CAA-RI, CAA-RI with isolated leptomeningitis, and CAA-RI without microbleeds (2–5). As the clinical spectrum of CAA-RI expands, diagnosis will continue to be challenging in some cases.

Only a small proportion of CAA patients develop vascular inflammation, the cause of which remain unclear. The apolipoprotein E (*ApoE*) genotype appears to play a role. It is well-known that the *ApoE* gene is closely related to sporadic CAA and sporadic Alzheimer's disease (AD). Although the ε2 allele has been considered to be “protective” against AD, it is a known vascular risk factor. Both the ε2 and ε4 alleles were found to be risk factors for CAA, spontaneous intracerebral hemorrhage and CAA-related hemorrhage, especially in Caucasian populations (6, 7). Current opinion is that ε2 can alter the vascular wall structure of CAA, thus making it more susceptible to hemorrhage (8, 9).

Few studies have assessed the association between *ApoE* genotypes and CAA-RI. They found that the frequency of the *ApoE* ε4/ε4 genotype was significantly higher in patients with CAA-RI than in those with CAA only (10). The presence of the *ApoE* ε4/ε4 genotype appears to be valuable in the diagnosis of CAA-RI.

The role of the *ApoE* ε2 allele in CAA-RI is largely unknown, partly due to the rarity of the ε2/ε2 genotype in the general population. To the best of our knowledge, this is the first report to describe the *ApoE* ε2/ε2 genotype in a patient with CAA-RI.

## case Report

A 69-years-old Chinese female farmer was admitted to the department of neurology due to cognitive decline and drooling during the previous month. She had difficulty remembering recent events, and the involuntary drooling was especially obvious during sleep. These symptoms gradually worsened. She experienced sleepiness, and became lost when outside alone. Mild urinary incontinence developed; however, no dizziness, headache, fever, limb numbness, or weakness were reported.

She had a 20-years history of hypertension with irregular use of reserpine, and her blood pressure was not well-controlled. She did not smoke or drink alcohol. There were no exposures to toxic substances or drugs. Family history was unremarkable for cognitive disorders or cerebral hemorrhages.

General physical examination was normal and, on neurological examination, she was alert. Although she exhibited fluent speech without comprehension difficulties, orientation, and calculation were impaired. Short-term memory declined. The cranial nerves were intact. Muscle strength and tone were normal and symmetric. Deep tendon reflexes were symmetrical and moderate, and coordinated movements were ably performed. No pathological reflex was elicited. Given that the patient was illiterate, she could not undergo detailed neuropsychological tests. She scored 18 points on the Mini-Mental State Examination (MMSE), with loss of orientation, 3-step command, calculation, clock drawing, and recall (cut-off value for Chinese illiterate dementia patients is < 19 points).

Routine blood tests revealed a mildly reduced platelet count of 64 × 10^9^/L (normal, 100–300 × 10^9^/L). Thyroid function, folic acid, and vitamin B_12_ levels were normal. Syphilis and HIV serological tests were negative. Serum paraneoplastic antibodies and anti-neuronal antibodies for autoimmune encephalitis were all negative. Tumor markers, including carcinoembryonic antigen, carbohydrate antigens 125 and 19–9, and alpha-fetoprotein, were within normal ranges. Serum TORCH-immunoglobulin (Ig) M antibody test was negative. Cerebrospinal fluid (CSF) examination revealed a normal cell count and protein level of 0.4 g/L (normal range, 0.15–0.45 g/L). No CSF oligoclonal IgG bands were detected. CSF levels of amyloid β (Aβ) 40 and Aβ42 examined using ELISA were lower than normal controls (Aβ40, 4,500 pg/ml, normal 6,400 pg/ml; Aβ42, 325 pg/ml, normal 500 pg/ml, in our lab).

**Figure 1:**
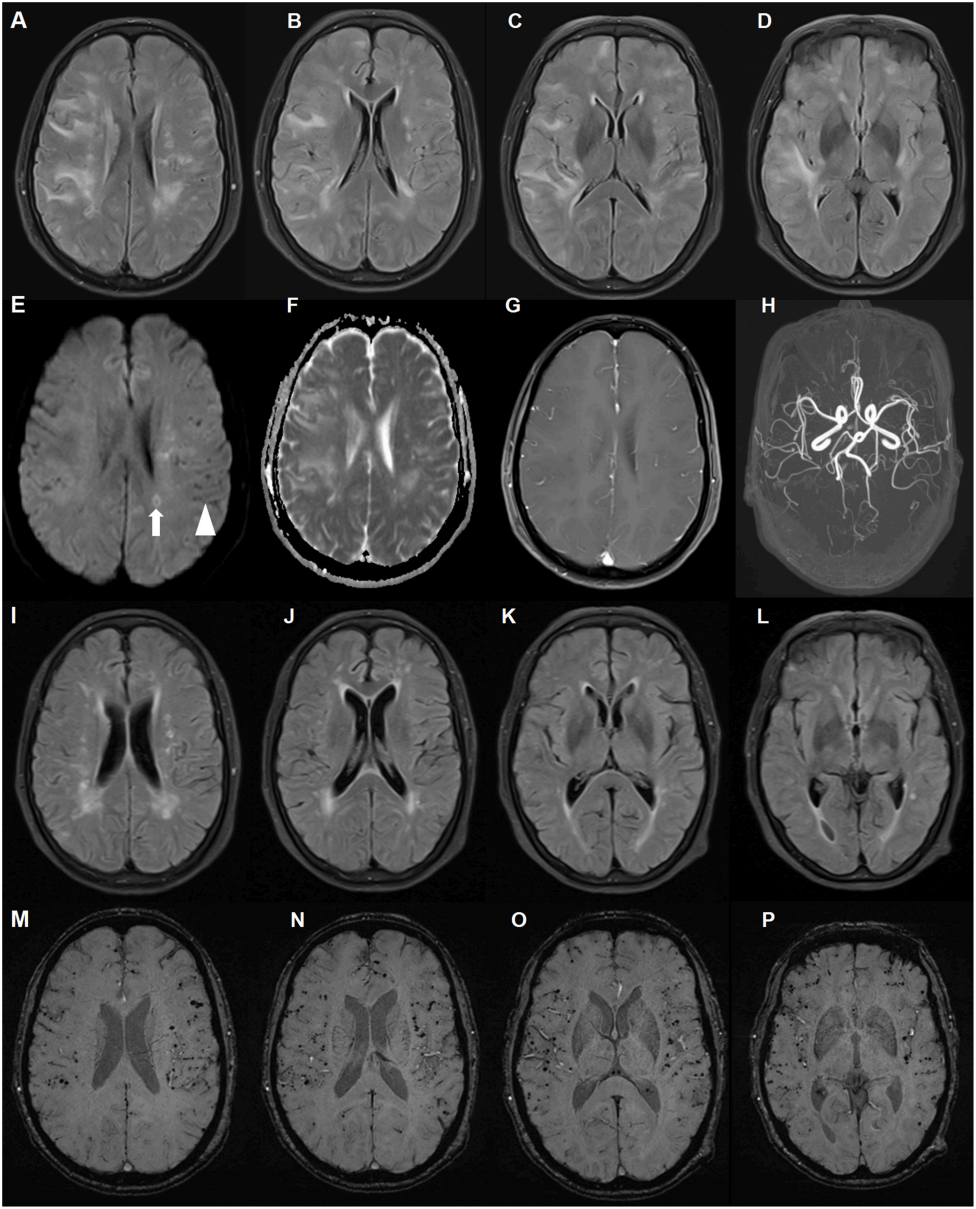
Brain magnetic resonance imaging (MRI) of a patient with cerebral amyloid angiopathy (CAA)-related inflammation (CAA-RI) with the *ApoE* ε2/ε2 genotype. Before glucocorticoid treatment, axial fluid attenuated inversion-recovery (FLAIR) **(A–D)** sequences revealing multiple, asymmetrical subcortical white matter lesions, predominantly in the left lobe. The lesions are isointense on diffusion-weighted imaging (DWI) sequence **(E)**, hyperintense on apparent diffusion coefficient (ADC) **(F)** and without parenchymal enhancement **(G)**. MRA did not reveal abnormalities **(H)**. Scattered hypointense lacunae (arrow head) and iso-hyperintense lesions (arrow) on the DWI sequence indicating microbleeds and subacute ischemia. On the follow-up MRI 2 months later, FLAIR lesions largely disappeared after glucocorticoid treatment **(I–L)**. Susceptibility weighted imaging revealed no changes in diffuse microbleeds **(M–P)**.

The patient underwent chest computed tomography, and abdominal, pelvic and breast ultrasound, which revealed no significant abnormalities. Initial brain magnetic resonance imaging (MRI) revealed multiple, asymmetrical subcortical white matter lesions, with U-fiber involved. These lesions were hyperintense on T2-weighted imaging, fluid attenuated inversion-recovery (FLAIR) and apparent diffusion coefficient (ADC) sequences, and were isointense on diffusion-weighted imaging (DWI) sequence. These neuroimaging findings were consistent with vasogenic edema. There was no parenchymal enhancement within the lesions. Other findings included chronic and subacute lacunar infarcts. Scattered hypointense lacunae were noted on DWI, while not prominent on other sequences. These lacunae suggested cerebral microbleeds. Subsequent susceptibility weighted imaging (SWI) confirmed diffuse microbleeds, mainly in the cortex (Figure 1). Although the patient had a long history of poorly controlled hypertension, deep cerebral microbleeds were largely absent. According to the revised clinico-radiological diagnostic criteria, she was diagnosed with CAA-RI based on clinical features and specific neuroimaging findings (11).

Due to the patient's concern about the side effects of large-dose glucocorticoids, she was administered intravenous methylprednisolone 120 mg daily for 5 days. Subsequently, 30 mg prednisone per day was administered with a 5 mg tapered reduction bi-weekly. Two months later, her memory improved and her MMSE score was 21 points. On follow-up brain MRI, diffuse white matter lesions largely improved with microbleeds unchanged (Figure 1), and prednisone was discontinued 1 month later. The most recent follow-up, 18 months since disease onset, revealed no clinical relapse.

Genomic DNA was extracted from peripheral blood leukocytes. *ApoE* genotypes were determined using polymerase chain reaction and direct sequencing. According to the different combinations of codon 112 and 158 of the fourth exon of the *ApoE* gene, the patient was found to harbor the rare ε2/ε2 genotype.

## Discussion

This case of CAA-RI presented with mild cognitive impairment without headache, epilepsy, or other focal neurological deficits. The MRI abnormalities were more severe than the clinical presentations. There were extensive microbleeds, but no lobar hemorrhage ever occurred. The diagnosis of CAA-RI in this patient was made without biopsy. Low Aβ levels in the CSF and good response to steroids supported the diagnosis of CAA-RI. Although primary angiitis of the central nervous system may have similar white matter lesions on brain MRI, diffuse cortical microbleeds are rare. Lymphoma lesions are also steroid-responsive on neuroimaging, but usually with prominent enhancement. There was no parenchymal enhancement within the lesion in the current case. The follow-up 18 months later did not support a diagnosis of lymphoma.

In CAA-RI, the *ApoE* ε4 allele and ε4/ε4 genotype are over-represented. Two previous studies found the *ApoE* ε4/ε4 genotype in 71–77% of CAA-RI patients, compared with only 4–5% of CAA patients without inflammation (10, 12). In one literature review, including 132 cases of CAA-RI, 22 of 36 (61%) with available *ApoE* genotype data harbored the ε4/ε4 genotype. The remaining *ApoE* genotypes were mainly ε3/ε3 (17%) and ε2/ε3 (17%) (13). Here, we report for the first time, a CAA-RI patient with the *ApoE* ε2/ε2 genotype.

*ApoE* ε2 has been found to be strongly linked to both CAA-related hemorrhage and spontaneous intracerebral hemorrhage, possibly by promoting vascular fibrinoid necrosis. One large neuropathological study found that either *ApoE* ε4 or ε2 could potentiate CAA pathology in both the parenchymal and meningeal vasculature (14). The ε2/ε2 genotype, similar to ε4/ε4, may also be a precipitating factor for CAA-RI. However, because the ε2 allele or genotype are relatively rare, it may be difficult to determine the role of ε2 in CAA-RI in studies with small sample sizes; thus, the role of ε2 in CAA-RI may be underestimated. To clarify the role of *ApoE* ε2 in CAA-RI, parallel studies aimed at determining whether ε2 is more common in patients with CAA-RI than in those with CAA only are warranted.

In summary, the present case was unique in two points. To our knowledge, this is the first report of a CAA-RI patient with the *ApoE* ε2/ε2 genotype. Moreover, the patient presented with mild clinical symptoms but striking neuroimaging features of CAA-RI. The response to small-dose glucocorticoids was satisfactory. As the rarest genotype, the role of *ApoE* ε2/ε2 in CAA-RI warrants further exploration.

## Ethics Statement

The case report has been performed in accordance with the ethical standards laid down in the 1964 Declaration of Helsinki and its later amendments. Written informed consent was obtained from the patient for the publication of this case report.

## Author Contributions

SC and J-WZ conceived the idea. Y-YX collected the clinical data. SC and J-HZ contributed to the writing of the manuscript. The revision of the manuscript was done by X-LC and J-WZ.

### Conflict of Interest Statement

The authors declare that the research was conducted in the absence of any commercial or financial relationships that could be construed as a potential conflict of interest.

## References

[1] Yamada M Itoh Y Shintaku M Kawamura J Jensson O Thorsteinsson L. Immune reactions associated with cerebral amyloid angiopathy. Stroke. (1996) 27:1155–62. 10.1161/01.STR.27.7.1155 8685920

[2] Kang P Bucelli RC Ferguson CJ Corbo JC Kim AH Day GS Teaching NeuroImages: cerebral amyloid angiopathy-related inflammation presenting with isolated leptomeningitis. Neurology. (2017) 9:e66–7. 10.1212/WNL.0000000000004218 28784645

[3] Liang JW Zhang W Sarlin J Boniece I Case of cerebral amyloid angiopathy-related inflammation - is the absence of cerebral microbleeds a good prognostic sign? J Stroke Cerebrovasc Dis. (2015) 4:e319–22. 10.1016/j.jstrokecerebrovasdis.2015.08.001 26341733

[4] Renard D Wacongne A Thouvenot E Radiologically isolated cerebral amyloid angiopathy-related inflammation. J Stroke Cerebrovasc Dis. (2017) 6:e218–20. 10.1016/j.jstrokecerebrovasdis.2017.08.002 28864038

[5] Banerjee G Alvares D Bowen J Adams ME Werring DJ. Minimally symptomatic cerebral amyloid angiopathy-related inflammation: three descriptive case reports. J Neurol Neurosurg Psychiatr. (2019) 90:113–5. 10.1136/jnnp-2017-317347 29535144 PMC6327917

[6] Tzourio C Arima H Harrap S Anderson C Godin O Woodward M. APOE genotype, ethnicity, and the risk of cerebral hemorrhage. Neurology. (2008) 70:1322–28. 10.1212/01.wnl.0000308819.43401.87 18256366

[7] Marini S Crawford K Morotti A Lee MJ Pezzini A Moomaw CJ. Association of apolipoprotein e with intracerebral hemorrhage risk by race/ethnicity: a meta-analysis. JAMA Neurol. (2019). 10.1001/jamaneurol.2018.4519. [Epub ahead of print]. 30726504 PMC6459133

[8] Rannikmae K Kalaria RN Greenberg SM Chui HC Schmitt FA Samarasekera N APOE associations with severe CAA-associated vasculopathic changes: collaborative meta-analysis. J Neurol Neurosurg Psychiatr. (2014) 5:300–5. 10.1136/jnnp-2013-306485 PMC4018226 24163429

[9] Rannikmae K Samarasekera N Martinez-Gonzalez NA Al-Shahi Salman R Sudlow CL Genetics of cerebral amyloid angiopathy: systematic review and meta-analysis. J Neurol Neurosurg Psychiatr. (2013) 4:901–8. 10.1136/jnnp-2012-303898 23457231

[10] Kinnecom C Lev MH Wendell L Smith EE Rosand J Frosch MP Course of cerebral amyloid angiopathy-related inflammation. Neurology. (2007) 8:1411–16. 10.1212/01.wnl.0000260066.98681.2e 17452586

[11] Auriel E Charidimou A Gurol ME Ni J Van Etten ES Martinez-Ramirez S Validation of clinicoradiological criteria for the diagnosis of cerebral amyloid angiopathy-related inflammation. JAMA Neurol. (2016) 3:197–202. 10.1001/jamaneurol.2015.4078 26720093

[12] Eng JA Frosch MP Choi K Rebeck GW Greenberg SM Clinical manifestations of cerebral amyloid angiopathy-related inflammation. Ann Neurol. (2004) 5:250–6. 10.1002/ana.10810 14755729

[13] Mendonca MD Caetano A Pinto M Cruz e Silva V Viana-Baptista M Stroke-like episodes heralding a reversible encephalopathy: microbleeds as the key to the diagnosis of cerebral amyloid angiopathy-related inflammation-a case report and literature review. J Stroke Cerebrovasc Dis. (2015) 4:e245–50. 10.1016/j.jstrokecerebrovasdis.2015.04.042 26142259

[14] Nelson PT Pious NM Jicha GA Wilcock DM Fardo DW Estus S APOE-ε2 and APOE-ε4 correlate with increased amyloid accumulation in cerebral vasculature. J Neuropathol Exp Neurol. (2013) 2:708–15. 10.1097/NEN.0b013e31829a25b9 PMC3715146 23771217

